# Discrete Element Framework for Determination of Sintering and Postsintering Residual Stresses of Particle Reinforced Composites

**DOI:** 10.3390/ma13184015

**Published:** 2020-09-10

**Authors:** Szymon Nosewicz, Jerzy Rojek, Marcin Chmielewski

**Affiliations:** 1 Institute of Fundamental Technological Research Polish Academy of Sciences, Pawińskiego 5B, 02-106 Warsaw, Poland; jrojek@ippt.pan.pl; 2 Łukasiewicz Research Network Institute of Electronic Materials Technology, Wólczyńska 133, 01-919 Warsaw, Poland; marcin.chmielewski@itme.edu.pl

**Keywords:** sintering, discrete element method, residual stress, particle-reinforced composites

## Abstract

In this paper, the discrete element method framework is employed to determine and analyze the stresses induced during and after the powder metallurgy process of particle-reinforced composite. Applied mechanical loading and the differences in the thermal expansion coefficients of metal/intermetallic matrix and ceramic reinforcing particles during cooling produce the complex state of stresses in and between the particles, leading to the occurrence of material defects, such as cracks, and in consequence the composite degradation. Therefore, the viscoelastic model of pressure-assisted sintering of a two-phase powder mixture is applied in order to study the stress field of particle assembly of intermetallic-ceramic composite NiAl/Al${}_{2}$O${}_{3}$. The stress evaluation is performed at two levels: macroscopic and microscopic. Macroscopic averaged stress is determined using the homogenization method using the representative volume element. Microscopic stresses are calculated both in the body of particles and in the contact interface (necks) between particles. Obtained results are in line with the cooling mechanism of the two-phase materials.

## 1. Introduction

Composite materials are an important class of advanced materials made from two or more components. A combination of the component properties and tailored microstructure allow obtaining a material with superior properties. The addition of reinforcement of particles to the composite matrix may lead to a considerable improvement of material properties, for example, the mechanical strength can be increased by the occurrence of strengthening mechanisms, such as Orowan strengthening, enhanced dislocation density, or load-bearing effect [1]. Due to their enhanced capabilities, particle-reinforced composites are attractive for many applications, including those sustained elevated temperatures. For example, considered here, an NiAl matrix composite reinforced with Al${}_{2}$O${}_{3}$ particles can be used instead of steel or metal alloys in aerospace and automobile industries for elements heated by intensive friction (brake discs, clutches, cranes, and valves) and for parts subjected to rapidly changing temperatures (nozzles, chambers combustion, engine guards, and exhaust systems) [2,3].

**Figure 1:**
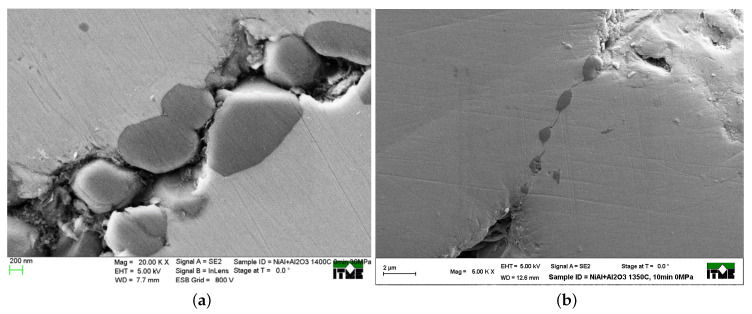
SEM images of NiAl/Al${}_{2}$O${}_{3}$ composite: (**a**) material failure on the particle boundary area and (**b**) ceramic distribution on the particle boundary of intermetallic particles.

Exposure to elevated temperatures generally has harmful effects on the mechanical properties of structural materials [4]. Transient or steady-state thermal stresses may cause material damage or failure. In intermetallic/metal matrix composites, there is generally a substantial mismatch between the coefficients of thermal expansion of the ceramic reinforcement and the metallic matrix. Thus, any temperature change will lead to thermal stresses, which can cause microcracks in the composite (Figure 1a) and affect the macroscopic mechanical properties of the composite material.

Thermal residual stresses in metal-ceramic composites constitute a problem due to their potentially detrimental consequences to material integrity in structural components that appeared already at the stage of material processing [5]. Metal and intermetallic matrix composite reinforced by ceramic particulate additions are mainly manufactured by powder metallurgy (PM) techniques. Hot pressing involving simultaneous powder compaction and sintering belongs to the most common PM techniques [6]. Macroscopic deformation and microstructural changes during pressure-assisted sintering in a hot pressing process occur under a complex state of stresses induced by sintering driving forces, applied pressure, and temperature changes. Some of the most frequent defects in sintered materials such as cracks and shape distortions are associated with stresses during and after the sintering process. Degradation of composite materials manufactured by powder metallurgy due to the progressive growth of microcracks (Figure 1a) is mainly induced by the difference in thermal expansion of the two interacting phases during the cooling stage. Residual stresses are accumulated in the areas of cohesive necks and interfaces. Small particle reinforcements in the necks between bigger matrix particles (Figure 1b) and irregularly shaped pores contribute to the concentration of thermal stress [7]. This shows that the determination of the stresses in the sintered composite material is an important practical problem. Knowledge of the stresses would be useful in the optimization of the material and manufacturing process, aiming at minimization of the danger of microcracking.

Modeling of stresses, defects, and, in consequence, damage of particle-reinforced composite materials induced by thermal loadings have been widely investigated, cf. [8,9,10,11,12]. Various approaches have been used in modeling of these phenomena. Deterioration of material properties due to high-temperature exposure can be considered using the continuum damage mechanics (CDM) approach incorporated in the finite element method (FEM) [13]. CDM models can be implemented within the elastic or elastoplastic framework, taking the material properties dependent on the particular damage variable, which in turn can be calibrated using experimental data. The modeling of thermal damage requires the coupling of thermal effects with mechanical damage. In the CDM model, a multiphase material, such as a particle-reinforced composite, is treated as homogeneous with certain effective properties. The effective macroscopic properties can be obtained experimentally or using some homogenization methods. Different homogenization methods developed to obtain effective properties of multiphase materials are reviewed in [14]. The simplest method is based on the rule of mixtures, which gives effective properties as volume average of respective phase properties. There are more sophisticated analytical methods such as the self-consistent method established by Eshelby [15] or Mori–Tanaka’s average stress theory [16]. These two classical methods enriched with interface debonding mechanisms have been used as theoretical bases of a mesoscopic constitutive model of particle-reinforced titanium matrix composites at high temperatures [17].

Different numerical homogenization methods use the finite element analyses of representative volume elements (RVE) to obtain effective properties of heterogeneous materials [18,19,20]. Understanding of a complex nonlinear response of multiphase materials at combined mechanical and thermal loading requires a model taking into account effects at the microlevel [21]. Therefore finite element models of RVEs intend to represent the material microstructure as exactly as possible. The development of X-ray micro-computed tomography (micro-CT) has made it possible to reconstruct a real composite microstructure which can be discretized with finite elements [22]. A real composite microstructure obtained from micro-CT has been used in the finite element analysis to obtain residual thermal stresses after manufacturing and effective properties of metal-ceramic composites [5,23].

An alternative numerical approach to finite element modeling is a discrete element method (DEM), which, in a simple and easy way, takes into account material defects existing or appearing during or after material processing or under thermal loading. Generally, the DEM was introduced for modeling granular materials in the pioneering works by Cundall [24,25] and Walton [26]. Recently, special DEM models are developed for powder metallurgy processes [27,28,29,30,31,32,33,34,35]. Most of the DEM applications for powder metallurgy have considered one-phase powder; there are very few works dealing with two-phase powders [36,37,38,39].

Moreover, the DEM enables simulation of materials stresses and, thus, fracture initiation and propagation [40]. The evolution of defects during the sintering of one-phase powder has been analyzed using the discrete element simulations by Martin et al. [41]. DEM has also been formulated for thermal and thermomechanical problems [42,43]. Discrete element simulation of thermal effects on the structure and properties of metal-matrix composites is rarely found any publication. A discrete element method to simulate thermal-induced damage in composite materials is presented in very recent publications [44,45].

The main objective of the present paper consists in numerical modeling of the stress response of a mixture of intermetallic and ceramic powder subjected to the uniaxial hot pressing process by taking into account phenomena occurring at microscopic and macroscopic scales. In respect to others papers in the related field, the proposed work is a first attempt focused on multiscale stress evaluation within discrete element framework performed in the context of the manufacturing process of particle-reinforced composites. In this paper, the intermetallic-ceramic composite NiAl/Al${}_{2}$O${}_{3}$ will be considered. The presented work demonstrates the possibilities of stress determination and analysis using the developed discrete element model of a powder metallurgy process [39]. The discrete element model considers the microstructure of the particle-reinforced composite and the phenomena associated with sintering, such as the influence of material densification on residual and thermal stresses during and after the powder metallurgy process.

## 2. Formulation of Discrete Element Framework

In the DEM, a material is represented by a large collection of particles interacting by contact forces. The translational and rotational motion of discrete elements (particles) are described by means of the Newton–Euler equations of rigid body dynamics. In principle, discrete elements can be of arbitrary shape; however, spherical particles are often a preferable choice because of the computational efficiency. The spherical discrete elements are also chosen for the present study.

The overall behavior of the system is determined by the contact laws assumed for the particle interaction [40]. The contact law can be seen as the formulation of the material model on the microscopic level. Contact models in the discrete element method can include force and moment interaction between particles. In the present work, contact moments are not considered. Below, contact laws for each stage of powder metallurgy process (compaction, heating, sintering, and cooling) have been presented briefly. The detailed description of the powder metallurgy model has been shown in [30,39]. The discrete element model developed according to the following formulation has been implemented in the discrete element code DEMpack [46].

### 2.1. Constitutive Contact Model of Compaction, Heating, and Cooling Stages

The discrete element contact model of the compaction process takes into account elastic deformation, viscous interaction, and friction at the contact point. The contact in normal direction is implemented using a Kelvin–Voigt-type model. The normal contact force $F_{n}$ is sum of the nonlinear elastic force $F_{n}^{e}$ and the viscous component $F_{n}^{d}$ [47]:

(1) $$F_{n}=F_{n}^{e}+F_{n}^{d}=\frac{4}{3}\bar{E}\sqrt{\bar{r}}{u_{\mathrm{rn}}^{e}}^{\frac{3}{2}}+c_{n}v_{\mathrm{rn}}$$

where $\bar{r}\left(T\right)$ is the effective radius of particle dependent on the temperature, $u_{\mathrm{rn}}^{e}$ is the particles penetration, $c_{n}$ is the coefficient of the viscosity, $v_{\mathrm{rn}}$ is the normal relative velocity, and $\bar{E}$ is the effective Young’s modulus defined as in [47]:

(2) $$\frac{1}{\bar{E}}=\frac{1-\nu_{i}^{2}}{E_{i}}+\frac{1-\nu_{j}^{2}}{E_{j}}$$

where $E_{a}$, and $\nu_{a}$ and a=i,j are the Young’s moduli and Poisson’s ratios of the contacting particles, respectively. The tangential contact force is evaluated assuming the regularized Coulomb friction model. Alternative contact models for powder compaction have been presented in [47].

A similar interaction with an additional cohesive interaction has been considered during cooling stage. Here, together with the heating and sintering stage, the process is treated as a thermomechanical problem. The uniform temperature has been prescribed in the whole body of particle assembly. Due to the small size of the studied sample (over 5000 particles), the heat conduction problem is neglected. One-way coupling between the thermal and mechanical problems is taken into account, resulting in the effect of thermal expansion of the particles (in consequence thermal stresses), and activation of the diffusion process started with the beginning of the sintering process.

### 2.2. Constitutive Contact Model of Sintering Stage

**Figure 2:**
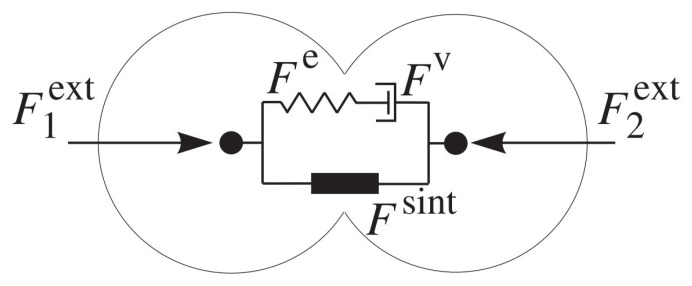
Rheological scheme of the viscoelastic model of pressure-assisted sintering.

The contact model for the sintering stage considers elastic and inelastic (viscous) deformation [39]. The viscoelastic component in this model is represented by a Maxwell-type element composed of the Hertzian nonlinear spring connected in series with the dashpot element representing the viscous-type interaction (Figure 2).

The form of the elastic force is the same as it shown in compaction model (Equation (1)). The viscous force is given by following relations [39],

(3) $$F_{n}^{v}=\eta v_{\mathrm{rn}}^{v}$$

where the viscosity coefficient η, following classical models of sintering developed at the particle level [48,49,50], can be expressed in terms of the effective grain boundary diffusion coefficient $D_{\mathrm{eff}}$ [28]:

(4) $$\eta =\frac{\pi a^{4}}{8D_{\mathrm{eff}}}$$

where *a* is the radius of cohesive neck. The effective grain boundary diffusion coefficient $D_{\mathrm{eff}}$ can be evaluated as follows [30],

(5) $$D_{\mathrm{eff}}=\frac{D_{\mathrm{gb}}\delta \Omega }{k_{B}T}$$

where Ω is the atomic volume, $k_{B}$ is the Boltzmann constant, *T* is the absolute temperature, and $D_{\mathrm{gb}}$ is the grain boundary diffusion coefficient with the width δ given by a Arrhenius-type equation [51]:

(6) $$D_{\mathrm{gb}}=D_{0\mathrm{gb}}\exp\left[-\frac{\Delta H_{\mathrm{gb}}}{RT}\right]$$

where $D_{0\mathrm{gb}}$ is the pre-exponential factor of grain boundary diffusion, $\Delta H_{\mathrm{gb}}$ is the activation enthalpy of grain boundary diffusion and *R* is the gas constant.

**Figure 3:**
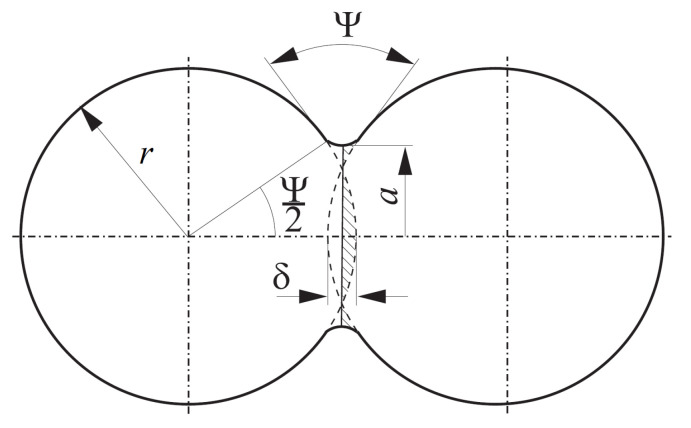
Two-particle model of sintering.

The sintering driving force $F_{n}^{\mathrm{sint}}$ results from surface tension on the particles grain boundary [52]:

(7) $$F_{n}^{\mathrm{sint}}=\pi \gamma_{S}\left[4\bar{r}\left(1-\cos\frac{\Psi }{2}\right)+a\sin\frac{\Psi }{2}\right]$$

where $\bar{r}$ is the effective particle radius, Ψ is the dihedral angle, $\gamma_{S}$ is the surface energy, and *a* is the neck radius of the interparticle boundary (Figure 3).

The maximum value of *a* indicating the end of sintering (the equilibrium state) can be described by the following geometric relationship [30].

(8) $$a_{\max}=r_{\min}\sin\frac{\Psi }{2}$$

As it was proved in [39], the interaction between real intermetallic NiAl particles with ceramic Al${}_{2}$O${}_{3}$ indicates an adhesive character of the contact bond. The viscoelastic model, presented above, assumes the cohesion between interacting discrete elements and good penetration of the particles resulting from the diffusive character of the contact bond. In the case of interface between NiAl and Al${}_{2}$O${}_{3}$ particles, the penetration of the particles depends mainly on the viscosity of the material and applied external pressure. Due to this fact, it has been assumed that the sintering driving force $F^{\mathrm{sint}}$, given by Equation (7), will be neglected in the model of interaction between the NiAl and Al${}_{2}$O${}_{3}$ particles during sintering.

### 2.3. Evaluation of Microscopic Stress

Microscopic observations of the fractures of the sintered material, such as in Figure 1, reveal fractures both in the body of single particles and/or in the necks between particles, named transgranular and intergranular fracture modes, respectively. This shows the necessity to consider the stresses both in the necks and in the particles.

#### 2.3.1. Microscopic Stresses in the Cohesive Necks

According to the two-particle sintering model presented in Section 2.2, the interaction force between particles is transmitted through the neck described by the circle cross-sectional area with a diameter of *a* (Figure 3). The total average stress σ in the neck during the sintering is the sum of the stress in the Maxwell element $\sigma^{\mathrm{ev}}$ and the induced by sintering driving force $\sigma^{\mathrm{sint}}$:

(9) $$\sigma =\sigma^{\mathrm{sint}}+\sigma^{\mathrm{ev}}$$

where

(10) $$\sigma^{\mathrm{sint}}=\frac{F_{n}^{\mathrm{sint}}}{A_{\mathrm{gb}}}$$

(11) $$\sigma^{\mathrm{ev}}=\frac{F_{n}^{e}}{A_{\mathrm{gb}}}=\frac{F_{n}^{v}}{A_{\mathrm{gb}}}$$

with $F^{\mathrm{sint}}$ being the sintering driving force, $F^{\mathrm{ev}}$—the force in the Maxwell element, and $A_{\mathrm{gb}}$—the cross-sectional area of the cohesive neck:

(12) $$A_{\mathrm{gb}}=\pi a^{2}$$

After the sintering process—$\sigma^{\mathrm{sint}}=0$—the residual stresses arise from the elastic component of the forces remaining after removing the load.

#### 2.3.2. Microscopic Stresses in the Particle Bodies

**Figure 4:**
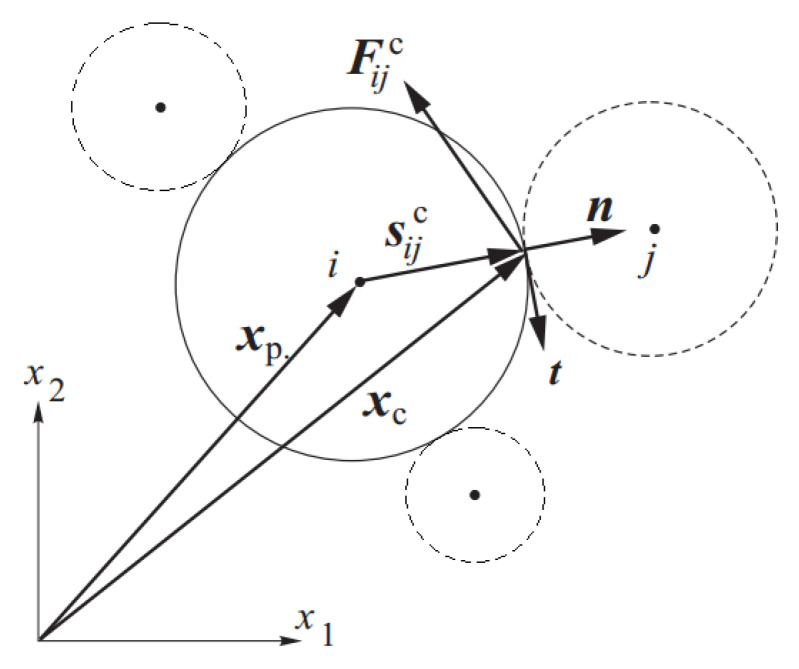
Definition of interparticle interaction [53].

Let us consider a particle surrounded by $n_{c}$ particles as it is shown in Figure 4.

The average stress $\sigma_{p}$ in the considered particle *i* is given by the following formula (cf. [54]),

(13) $$\sigma_{p}=\frac{1}{V_{p}}\sum_{j=1}^{n_{i}^{c}}s_{ij}^{c}⊗F_{ij}^{\;c}$$

where $V_{p}$ is the element volume, $s_{ij}^{c}$—vector connecting the element center with the contact point, $F_{ij}^{\;c}$—the contact force between the particles *i* and *j*, and the symbol ⊗ denotes the outer (tensor) product. The contact force $F_{ij}^{\;c}$ and the vector $s_{ij}^{c}$ are shown in Figure 4.

As the particle in a general case is not in equilibrium the tensor $\sigma_{p}$ obtained from Equation (13) can be non-symmetric. It can be symmetrized as follows,

(14) $$\sigma_{p}^{\mathrm{sym}}=\frac{\sigma_{p}+\sigma_{p}^{T}}{2}$$

Furthermore, the stress tensor $\sigma_{p}^{\mathrm{sym}}$ can be decomposed into the deviatoric and hydrostatic (mean) stress—$\sigma_{p}^{\mathrm{dev}}$ and $\sigma_{p}^{\mathrm{hyd}}$:

(15) $$\sigma_{p}^{\mathrm{sym}}=\sigma_{p}^{\mathrm{dev}}+\sigma_{p}^{\mathrm{hyd}}=\sigma_{p}^{\mathrm{dev}}+I\sigma^{m}$$

where $\sigma^{m}$ is the mean stress

(16) $$\sigma^{m}=\frac{\sigma_{xx}+\sigma_{yy}+\sigma_{zz}}{3}$$

and $\sigma_{xx},\sigma_{yy},\sigma_{zz}$ are the components of the stress tensor. The hydrostatic stress given by Equation (16) will be used later as a suitable parameter to characterize if the particle is subjected to tension or compression.

### 2.4. Evaluation of Macroscopic Stress

Effective macroscopic variables and properties in micromechanical models can be determined by various analytical and numerical homogenization and averaging methods [55,56,57,58,59,60]. In this work, averaging methods based on the concept of the representative volume element (RVE) will be used [61,62]. Given constant (averaged) stresses in particles expressed by Equation (13), the average stress in the representative volume element can be calculated as (cf. [54,63])

(17) $$\bar{\sigma }=\frac{1}{V_{\mathrm{RVE}}}\sum_{p\in V_{\mathrm{RVE}}}V_{p}\sigma_{p}=\frac{1}{V_{\mathrm{RVE}}}\sum_{p\in V_{\mathrm{RVE}}}\sum_{j=1}^{n_{i}^{p,c}}s_{ij}^{c}⊗F_{ij}^{\;c}$$

The expression (17) for the average stress over the representative volume can be written in an alternative equivalent form (cf. [63]):

(18) $$\bar{\sigma }=\frac{1}{V_{\mathrm{RVE}}}\sum_{c=1}^{N_{c}}L^{c}⊗F^{c}$$

in which summation is over all $N_{c}$ contacts in the representative volume element; $F^{c}$ is the total contact force for each contact; $V_{\mathrm{RVE}}$ is the volume of RVE; and $L^{c}$ is the so-called branch vector connecting the centroids of two particles, *i* and *j*, defined as follows,

(19) $$L^{c}=x_{p}^{\left(i\right)}-x_{p}^{\left(j\right)}$$

Tensor $\bar{\sigma }$ can be symmetrized analogously to Equation (14). Equation (18) will be used later in this work to calculate macroscopic stresses taking the whole specimen as the representative volume element.

## 3. Numerical Results

### 3.1. Calibration of Discrete Element Model of Powder Metallurgy

The discrete element model of the pressure-assisted sintering of a two-phase NiAl/20%Al${}_{2}$O${}_{3}$ powder mixture has been calibrated and validated by Nosewicz et al. in [39]. The methodology of simulation set-up, the procedure of evaluation of material parameters of sintering model, and the final comparison with experimental results of density evolution have been described widely.

**Figure 5:**
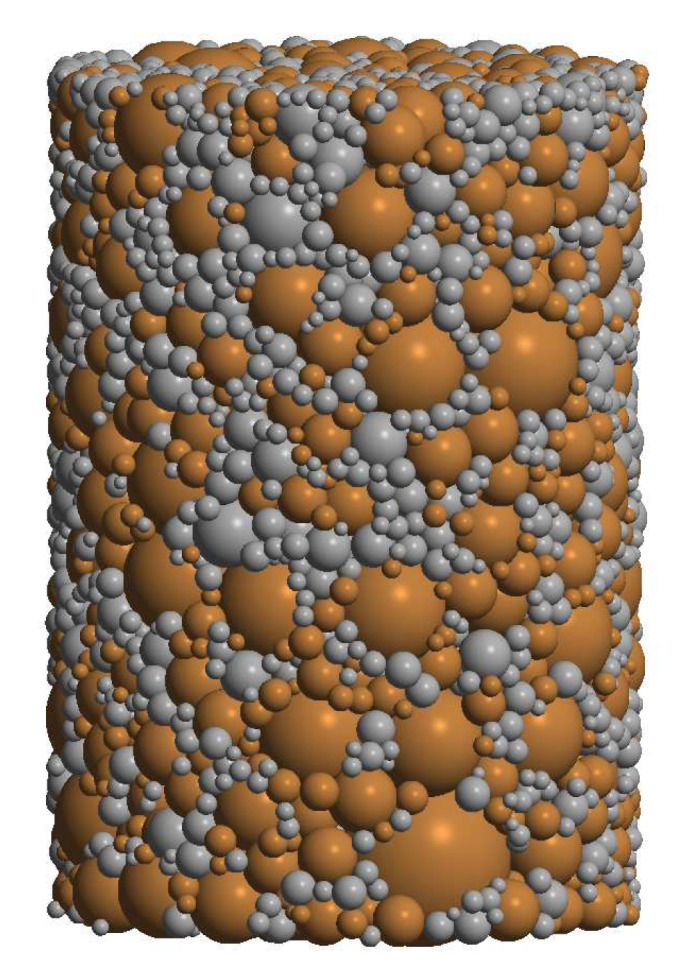
Discrete element specimen of two-phase 80%NiAl-20%Al${}_{2}$O${}_{3}$ powder.

The discrete element geometrical assembly has been obtained (Figure 5) taking the particle size as the same as in the real powder using a specially designed procedure [30]. The DEM specimen generated with this algorithm satisfies the main requirements, such as material isotropy, uniform distribution of reinforcement (ceramic) particles in intermetallic NiAl matrix, the irregular configuration of particles, real particle size distributions, relatively low porosity, and dense packing of powder particles.

The simulation of a two-phase powder mixture assumes three types of material interaction: between matrix particles, between reinforcement particle, and mixed matrix-reinforcement. The materials parameters of the sintering model for each contact have been estimated (and fitted) on the basis of equations and relations [30]. The full set of parameters are shown in Table 1.

**Figure 6:**
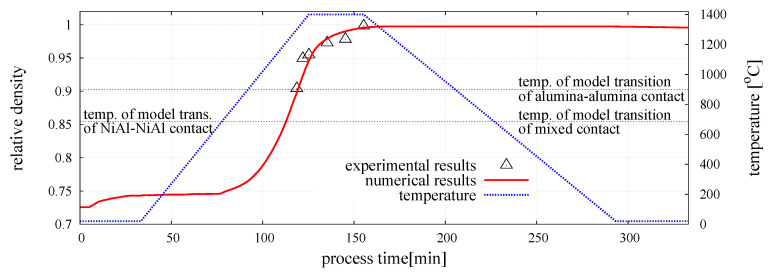
Evolution of the relative density of the mixture of NiAl–Al${}_{2}$O${}_{3}$ powder [39].

The sintering model of the two-phase material has been calibrated for the following sintering process parameters; external pressure p=30 MPa, sintering temperature $T_{s}=1400$ ${}^{{}^{\circ}}C$ (1673 K), and sintering time $t_{s}=30$ min. The run of numerical and experimental results of the relative density of the sintered NiAl-20%Al${}_{2}$O${}_{3}$ [64] specimen has been plotted in Figure 6 together with the temperature profile. Adequate correspondence between numerical and experimental results confirms a correct performance of the presented model. The discrete element modeling of the powder metallurgy process has allowed us to obtain a numerical representation of sintered specimens for a certain combination of sintering process parameters.

### 3.2. Evolution of the Macroscopic Stress

**Figure 7:**
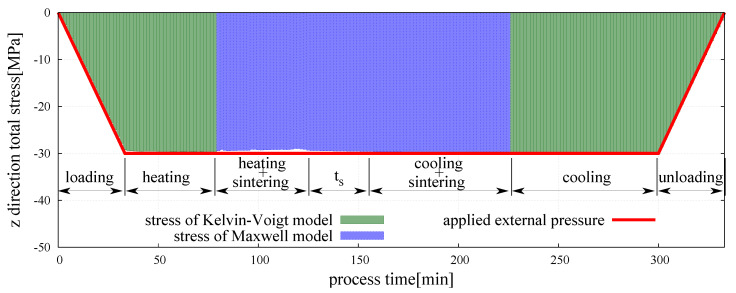
Evolution of total macroscopic stress of the hot pressing process in *z* direction.

Stress analysis has been performed for manufacturing of the composite specimen NiAl/20%Al${}_{2}$O${}_{3}$ with the same sintering process parameters as presented in Section 3.1. The total $\bar{\sigma }$, sintering driving ${\bar{\sigma }}^{\mathrm{sint}}$, and viscoelastic ${\bar{\sigma }}^{\mathrm{ev}}$ macroscopic stresses have been calculated from Equation (18) using the corresponding force components $F_{n}$, $F_{n}^{\mathrm{sint}}$, and $F_{n}^{e}$, respectively. Figure 7 shows the evolution of the total macroscopic stress in the composite specimen during the whole powder metallurgy process. The intervals corresponding to each stage—loading, heating, sintering, cooling, and unloading—have been shown in order to enable a better understanding of stress changes.

**Figure 8:**
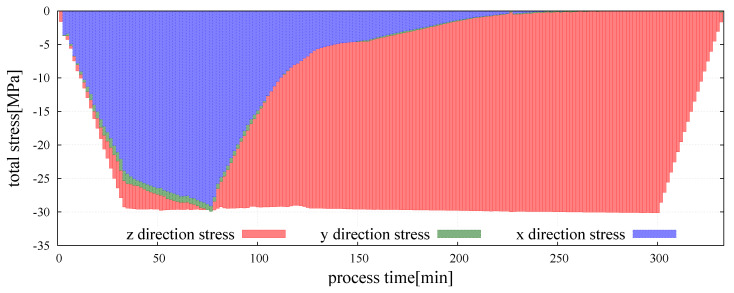
Evolution of total macroscopic stress of the hot pressing process in *x*, *y*, and *z* directions.

The graph presents the evolution of total macroscopic stress in *z* direction, in which the external pressure was applied. A comparison of the evolution of total macroscopic stress in *x*, *y* and *z* directions, respectively, ${\bar{\sigma }}_{\mathrm{xx}}$, ${\bar{\sigma }}_{\mathrm{yy}}$ and ${\bar{\sigma }}_{\mathrm{zz}}$, is presented in Figure 8.

**Figure 9:**
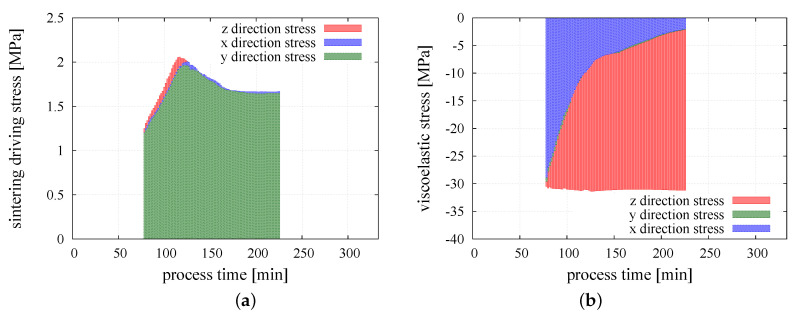
Evolution of (**a**) sintering driving macroscopic stress; (**b**) viscoelastic macroscopic stress of the sintering process in *x*, *y*, and *z* directions.

At the beginning of powder metallurgy process, the external pressure is applied and the total macroscopic stress of the composite in *z* direction arises to reach the value close to 30 MPa over a dozen minutes. At this moment, the proceed material is in mechanical equilibrium—the total macroscopic stress is equal to the value of the external pressure. After this point, a very fine variation of total macroscopic stress due to the effect of thermal expansion resulting from the increase of temperature can be seen. As the temperature of sintering activation for each material contact type is reached, the transition from the Kelvin–Voigt model to the sintering model occurs and the sintering is started. The beginning of sintering stage is accompanied by the appearance of the resultant force of sintering driving stress which acts in parallel with viscoelastic force in the Maxwell branch. The evolution of the sintering driving and viscoelastic stresses in the three normal directions of particle contact is presented in Figure 9a,b, respectively.

**Figure 10:**
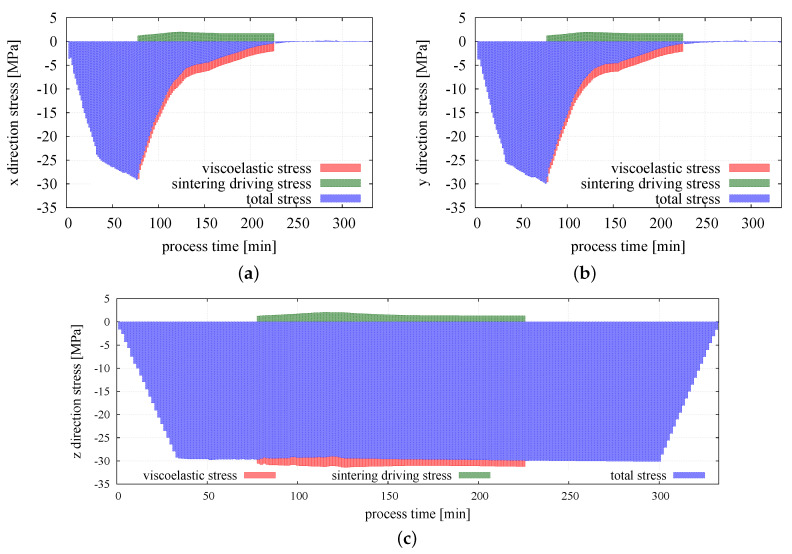
Evolution of the total sintering of the driving and viscoelastic macroscopic stresses: (**a**) in the *x* direction, (**b**) in the *y* direction, and (**c**) in the *z* direction.

Sintering driving stress in all directions is equal, which can be expected as the sintering driving stress should be an isotropic field. This confirms the correct performance of the model. Comparing to the magnitude of the external pressure, the value of the sintering driving stress is relatively small. The maximum value of the sintering driving stress, 2.05 MPa, corresponds to 6% of the value of the applied external pressure. The obtained values are consistent with the literature reports on theoretical and numerical analysis of sintering driving stress [65,66]. Macroscopic stress resulting from the sintering driving force has a positive value and together with applied external load is responsible for material densification and decreasing the porosity of the sintered powder. The material resistance is manifested by the viscoelastic macroscopic stress. Evolution of the three principal viscoelastic macroscopic stresses is shown in Figure 9b. It can be seen that before sintering, all three stress components are nearly equal, which is expected as the state of hydrostatic compression have been applied. With the progress of sintering, the radial stresses gradually decrease nearly to zero due to radial shrinkage of the specimen, and finally the state of uniaxial compression is obtained. In the middle and final stages of sintering, the viscoelastic macroscopic stress in *z* direction stabilizes around the value of 30 MPa, which indicates the balances of material resistance with external pressure and sintering driving stress. The graphical representation of total, viscoelastic, and sintering driving macroscopic stress in three main directions for the whole period of the simulation is presented in Figure 10a–c.

The values of the principal macroscopic stresses at the final stage of sintering (t${}_{s}=30$ min) and after unloading are given in Table 2. As it is expected, the macroscopic stresses after the process are nearly zero. A very small value obtained because the equilibrium of the particles is not perfect. It should be remarked, however, that zero macroscopic stresses do not signify that microscopic residual stresses are also zero—it only means that microscopic residual stresses are self-equilibrated. Microscopic stress distribution will be investigated below.

### 3.3. Microscopic Stress in the Cohesive Necks

**Figure 11:**
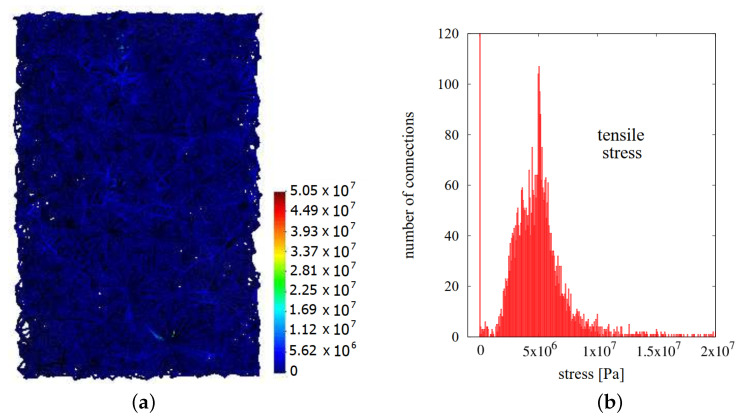
The results of sintering driving microscopic stresses at the final stage of sintering: (**a**) distribution in the volume and (**b**) histogram.

**Figure 12:**
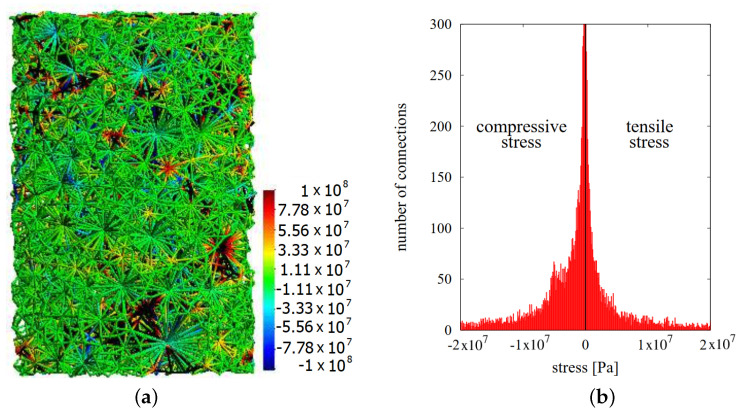
The results of viscoelastic microscopic stresses at the final stage of sintering: (**a**) distribution in the volume and (**b**) histogram.

Microscopic stresses in the necks have been determined for the case presented in Section 3.2. The stresses have been evaluated for all active interparticle cohesive necks, thus for those stresses for which neck radius *a* has not achieved an equilibrium state yet. Figure 11a and Figure 12a present composite specimens during hot pressing with the network of cohesive bonds represented by beams connecting the centers of interacting particles. Using Equations (9)–(11), the total σ, sintering driving $\sigma^{\mathrm{sint}}$, and viscoelastic $\sigma^{\mathrm{ev}}$ microscopic stresses have been calculated.

Microscopic stresses have been determined at the two special moments of hot pressing process: at the end of sintering time (t${}_{s}=30$ min) and after cooling and unloading. Figure 11 presents the distribution and histogram of sintering driving microscopic stresses in the final stage of sintering (before material cooling). Similarly, Figure 12 shows the results of viscoelastic microscopic stresses.

As it was presented in the previous paragraph, the value of sintering driving stress is rather insignificant compared to the total stress. The mean value of the sintering driving microscopic stress is ~2 MPa, and most of connections are in the range from 0 to 10 MPa. Sintering driving microscopic stress affects the attraction of particles; however, in the view of the above, the applied external pressure has a more crucial impact on the powder densification.

Attractive contact interaction resulting from the impact of sintering driving and external stress is balanced by the viscoelastic resistance of the material. Analyzed final stage of sintering is characterized by the equilibrium of sintered material, where the motion of particles in the contact is the minimum, and practically material compaction does not occur. The viscoelastic microscopic stress has both positive and negative values; thus, it indicates the tensile and compressive character of interaction (Figure 12b).

Furthermore, it can be seen the advantage of the compressive stresses (negative values) over the tensile ones (positive), which is related to material resistance from the external pressure application. It should also be noted that in specific locations of the composite specimen, viscoelastic microscopic stresses achieve significant values, both tensile and compressive—even around 52 GPa. Naturally, the obtained results of maximum and minimum microscopic stresses are unrealistic and result from no occurrence of cracking model of interacting particles.

**Figure 13:**
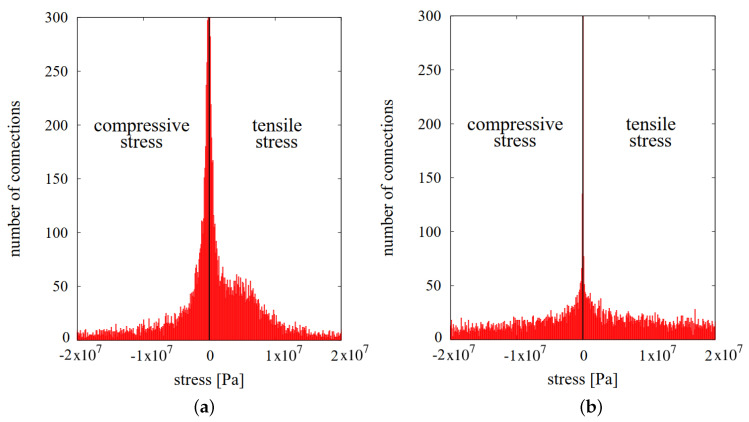
Histograms of total microscopic stresses: (**a**) in the final stage of sintering and (**b**) after sintering (residual microscopic stresses).

Due to the model assumptions and following Equation (9), viscoelastic stress is the major component of total microscopic stress in the Maxwell element. Figure 13 introduces the comparison of histograms of total microscopic stress during (the final stage of sintering) and after the hot pressing process (residual stresses). The properties of composite material after the process (after the cooling), compared to properties during the sintering, are various due to no more effect of temperature and compressive pressure. After the sintering, the specimen is subject to other conditions, which is reflected in the different form of the microscopic stress distribution.

Total microscopic stress (Figure 13a) is the sum of sintering driving stress (Figure 11b) and viscoelastic stress (Figure 12b). The combination of tensile stresses (impact of sintering driving and viscoelastic stress) and compressive stresses (impact of viscoelastic stress as the effect of the response of the material to applied external force) can be seen. In the second case (Figure 13b), after hot pressing, the only interaction is the viscoelastic one. Sintering driving stress is deactivated (no temperature effect—sintering driving force equal zero) and the loading of punch is removed (external force equal zero). In this case, the residual microscopic stresses indicate the compressive and tensile character and similar values, which proves that a sintered composite specimen occurs in the stress equilibrium.

### 3.4. Microscopic Stress in the Particles

**Figure 14:**
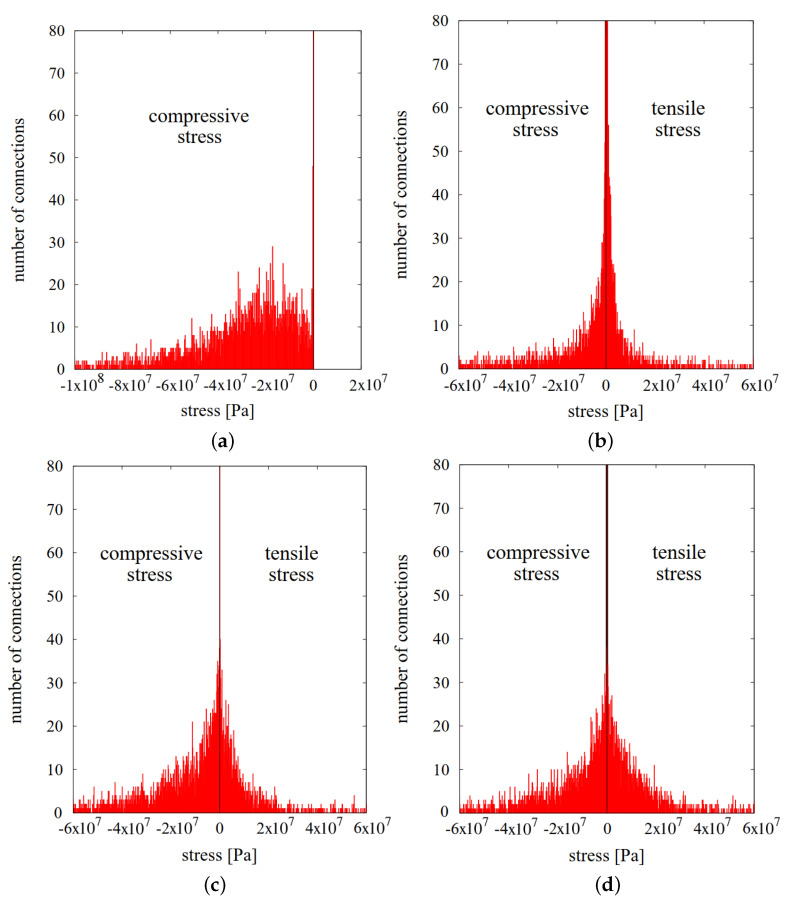
Distribution of particle total hydrostatic stresses: (**a**) after loading, before heating (t = 33 min); (**b**) in the final stage of sintering (t = 155 min, t${}_{s}=30$ min); (**c**) after cooling, before unloading (t = 293 min); and (**d**) after unloading.

Averaged microscopic stresses in the particle bodies have been evaluated during and after the hot pressing process investigated in the previous sections. The total microscopic stress in each discrete element was calculated from Equation (13). In order to compare the stresses at specific stages of the hot pressing, the histograms of the microscopic stresses of the whole volume of the NiAl/Al${}_{2}$O${}_{3}$ specimen have been presented. Figure 14a–d shows the stress distributions at the four stages: after loading (before heating), in the final stage of sintering, after cooling (before unloading), and finally after unloading, respectively.

**Figure 15:**
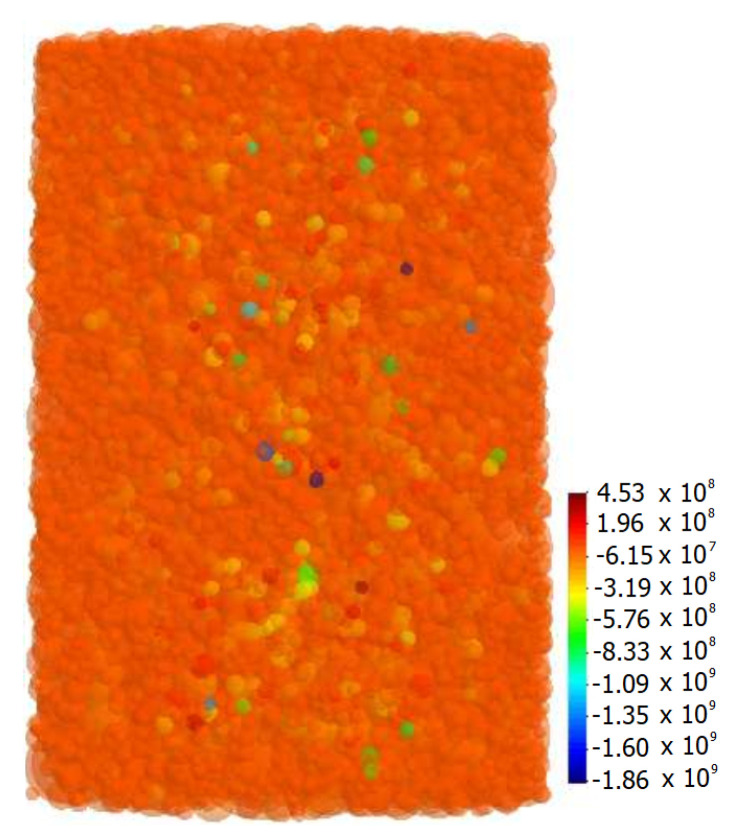
Graphical distribution of particle total hydrostatic stresses in the final stage of sintering.

In the first case, it can be seen the material response to the external pressure in the form of compressive stresses. The stress distribution is changed after sintering activation (Figure 14b). The sintering stage is characterized by the occurrence of sintering driving stresses and a combination of viscoelastic tensile and compressive stresses. Sintering driving stresses are responsible for discrete element attraction and indicate the tensile character, which can be seen in Figure 11b. The values of sintering driving microscopic stress range from 0 to 10 MPa and are insignificant comparing to the viscoelastic ones. Furthermore, the cohesive particle interaction highly affects the maximum and minimum values of stresses, which was presented in Table 3. The graphical distribution of the maximum and minimum values of particle total microscopic stresses can be seen in Figure 15.

Presented figure leads to the obvious conclusion that the large local stresses are mostly generated in the smallest particles. The smallest particles are mainly subjected to cracking both during the sintering and cooling. As in the case of neck microscopic stress analysis, microscopic stress of particle achieves huge compressive values. Everything points that the application of the cracking model may decrease these large values of maximum and minimum microscopic stresses and provide more realistic analysis.

As the sintering ends and the specimen is cooled, sintering driving microscopic stress is deactivated, and the only particle interaction is the viscoelastic one. This stage of the process is similar to the first considered one (after loading, before cooling); however, the sintering has affected the specimen microstructure by creating the cohesive necks. Due to this fact, both compressive and tensile stress had been generated, however, the compressive ones prevail. Moreover, the maximum and minimum value of stress furthermore increased during the cooling stage because of the effect of thermal expansion.

After unloading, at the end of the process, the values of residual microscopic stress indicate the balance between positive (tensile) and negative (compressive) values of microscopic stresses.

**Figure 16:**
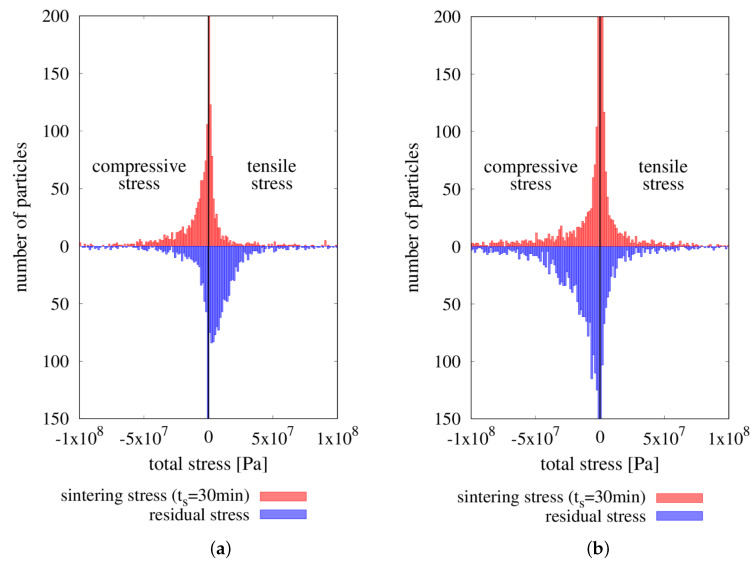
The histograms of particle total hydrostatic stresses in the final stage of sintering and at the end of the process (after cooling) of (**a**) NiAl and (**b**) alumina particles.

The further analysis concerns the microscopic stress determined and analyzed separately for the intermetallic NiAl and ceramic Al${}_{2}$O${}_{3}$ particles. The comparison of the results of obtained microscopic stresses of each phase is presented in Table 4. Figure 16 presents the histograms of microscopic stress of each phase in the final stage of sintering and after the unloading.

The values of microscopic stresses in the final stage of sintering of both materials, NiAl and Al${}_{2}$O${}_{3}$, are predominantly negative (compressive), which is consistent with the histograms in Figure 16, where compressive stresses predominate. In turn, the state of unloading and after cooling is characterized by different signs of the mean stresses of interacting phases. The mean value of the microscopic stress in the NiAl particles is positive, which indicates the tensile stress in the intermetallic particles. A negative value of the mean value of the microscopic stress in the Al${}_{2}$O${}_{3}$ particles indicates the compressive state of stress. Moreover, the unfavorable impact of residual thermal stresses can be seen in the extreme values. Comparing with the state of stress at the final stage of sintering, the maximum (tensile) stress grows in the case of the intermetallic phase and the minimum (compressive) stress increases in the case of alumina particles.

**Figure 17:**
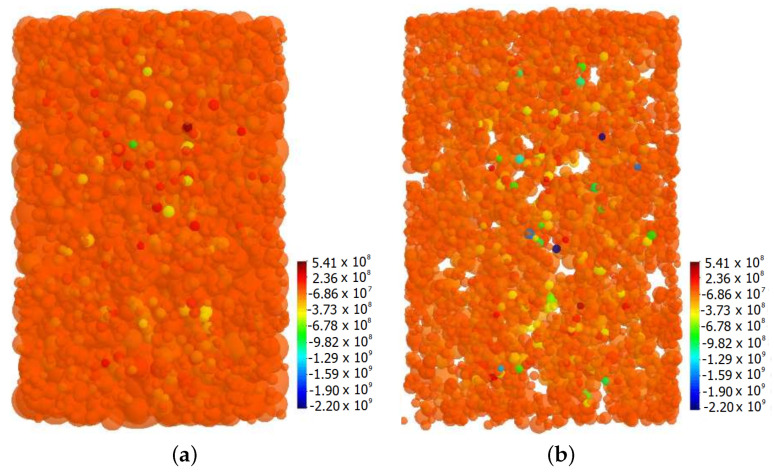
Graphical distribution of particle total hydrostatic stresses at the end of the process (after cooling) of (**a**) particles of intermetallic NiAl and (**b**) particles of ceramic Al${}_{2}$O${}_{3}$.

The presented results are in agreement with theoretical predictions regarding the cooling mechanism of the composite material. The differences in the coefficients of thermal expansion of two-phase material produces the effect of compression of the particles with a lower ability for shrinkage during the cooling. Ceramic particles shrink less, whereas the intermetallic ones, with a higher value of the coefficient of thermal expansion, deform more. Therefore, the intermetallic particles compressed the ceramic particles, introducing the large states of stress in the particle contacts. The presented contact interaction between the intermetallic and ceramic particles induces the occurrence of a state of stress, which can overweight the strength of the particles and consequently leads to the formation of the microcracks. Generally, in the ceramic phase of the metal-ceramic composite, compressive stresses prevail, but large local tensile stresses may also appear [23]. Ceramic particles are sensitive to microcracking due to the insignificant tensile strength. As it is shown in Figure 17, in spite of the mostly small alumina particles achieves the large values of compressive stresses, the significant tensile stresses can be also found.

### 3.5. Conclusions

An evaluation of stresses in the discrete element model of a powder metallurgy process has been presented. The knowledge of residual stresses is important for the design of the composite material and optimization of the manufacturing process, aiming to minimize the risk of possible material defects. Stress analysis has been performed for the selected case of the manufacturing of the NiAl-Al${}_{2}$O${}_{3}$ composite specimen. The stresses during and after the powder metallurgy process have been investigated at two levels of material scale: micro- and macroscopic. Microscopic stress has been determined and analyzed concerning two particular places subject to stress generation: necks between the powder particles and the whole body of particles. The results were analyzed considering different kinds of microscopic stresses: total, viscoelastic, and sintering driving.

Presented results are in agreement with theoretical predictions referred to the material states during the hot pressing process. During the sintering, the material is subjected to the applied external pressure and sintering driving stress indicating the combination of tensile stresses (impact of sintering driving and viscoelastic stress) and compressive stresses (impact of viscoelastic stress as the effect of material response to applied external force). In the case of the unloaded and cooled specimen, the compressive and tensile microscopic stresses show similar values, which has evidenced a state of equilibrium in the composite material.

In order to study the microscopic stress generated at each phase, the stresses have been determined separately for the intermetallic NiAl and ceramic Al${}_{2}$O${}_{3}$ particles. The numerical model predicts correctly that ceramic particles are compressed by intermetallic particles during the cooling stage due to the various values of the coefficients of thermal expansion. Comparing to the state of stress at the sintering stage, the maximum (tensile) stresses grow more in the case of the intermetallic phase, and the minimum (compressive) stresses increase more in the case of alumina particles.

Numerical averaging methods have been employed to determine the macroscopic stress of particle-reinforced composite material. Macroscopic stresses have been calculated for the whole process including loading, heating, sintering, cooling and unloading. The obtained results have confirmed the correct and efficient performance of the proposed numerical model. It has been found out that the macroscopic stresses are consistent with changing sintering process parameters.

## Figures and Tables

**Table 1:** Materials model parameters of different contact interactions.

Material Constant	Parameter Value		
	NiAl–NiAl	Al${}_{2}$O${}_{3}$–Al${}_{2}$O${}_{3}$	NiAl–Al${}_{2}$O${}_{3}$
Mean atomic volume, Ω [m${}^{3}$]	$1.20\times 10^{-29}$	$8.47\times 10^{-30}$	$9.01\times 10^{-30}$
Pre-exponential factor of the
grain boundary diffusion, $D_{0\mathrm{gb}}$ [m${}^{2}/$s]	$2.55\times 10^{-5}$	9.751	$3\times 10^{-2}$
Activation enthalpy of
grain boundary diffusion, $\Delta H_{\mathrm{gb}}$ [kJ/mol]	185	389	280
Grain boundary width, δ [nm]	0.5	0.5	0.5
Dihedral angle, Ψ [${}^{\circ }$]	147	127	135
Young’s modulus, *E* [GPa]	183	404	-
Poisson’s ratio, ν	0.34	0.232	-
Surface energy, $\gamma_{s}$ [J/m${}^{2}$]	1.57	1.28	-
Density, $\rho_{\mathrm{theo}}$ [kg/m${}^{3}$]	5910	3970	-
Coefficient of thermal expansion, α [$10^{-6}$ K${}^{-1}$]	11.5	7.4	-

**Table 2:** Summary of macroscopic stresses in three main directions at the final stage of sintering and the end of powder metallurgy process [MPa].

	Final Stage of Sintering − t${}_{s}=30$ min			End of the Process
Direction/Stress	Sintering Driving Stress	Viscoelastic Stress	Total Stress	Residual Total Stress
XX	1.8	−6.1	−4.3	−0.00006
YY	1.8	−6.3	−4.5	−0.000001
ZZ	1.5	−31.1	−29.6	−0.000075

**Table 3:** Evolution of maximum and minimum values of particle hydrostatic stresses [MPa] during the hot pressing process.

	Hot Pressing Stages			
	After Loading	Final Stage of Sintering	After Cooling	After Unloading
	t = 3 3 min	t = 155 min	t = 293 min	t = 330 min
maximum value	0.2	452.6	532.3	540.6
minimum value	−463.4	−1861.6	−2292.3	−2201.2

**Table 4:** Statistical parameters of particle total hydrostatic stresses [MPa] of each phase in NiAl/Al${}_{2}$O${}_{3}$ specimen.

Parameter/Stress	Final Stage of Sintering		End of the Process	
	NiAl	Al${}_{2}$O${}_{3}$	NiAl	Al${}_{2}$O${}_{3}$
mean value	−10.3	−16.1	1.9	−25.3
standard deviation	51.6	95.7	53.6	110.6
t maximum value	374.2	452.6	540.6	387.4
minimum value	−814.3	−1861.6	−806.5	−2201.2
